# Frequency of Immune Checkpoint Inhibitor-Induced Vasculitides: An Observational Study Using Data From the Japanese Adverse Drug Event Report Database

**DOI:** 10.3389/fphar.2022.803706

**Published:** 2022-03-25

**Authors:** Koki Kato, Tomohiro Mizuno, Takenao Koseki, Yoshimasa Ito, Kazuo Takahashi, Naotake Tsuboi, Shigeki Yamada

**Affiliations:** ^1^ Department of Clinical Pharmacy, Fujita Health University School of Medicine, Toyoake, Japan; ^2^ Department of Nephrology, Fujita Health University School of Medicine, Toyoake, Japan; ^3^ Department of Biomedical Molecular Sciences, Fujita Health University School of Medicine, Toyoake, Japan

**Keywords:** immune checkpoint inhibitor, polymyalgia rheumatica (PMR), Japanese adverse drug event report (JADER), adults, vasculitides

## Abstract

Information on immune checkpoint inhibitor-induced vasculitides is limited, and predictors for this condition have not been identified. Therefore, we have examined the frequency of immune checkpoint inhibitor-induced vasculitides by analyzing the data recorded in the Japanese Adverse Drug Event Report database. Data from April 2004 to March 2020 were extracted, and vasculitides as an immune-related adverse event was defined according to the 2012 revised International Chapel Hill Consensus Conference Nomenclature of Vasculitides. Adverse event signals were recognized as significant when the reporting odds ratio estimates and lower limits of the corresponding 95% confidence intervals exceeded 1. The use of nivolumab showed a significant signal for vasculitides. Furthermore, significant signals of polymyalgia rheumatica were found when the patients were treated with nivolumab, pembrolizumab, and ipilimumab. In addition, the frequencies of nivolumab- and pembrolizumab-induced polymyalgia rheumatica were higher in patients aged ≥70 years and female patients, respectively. Polymyalgia rheumatica was reported in 38 patients treated with nivolumab; 31 (82%) of these were either in recovery or in remission. Further, polymyalgia rheumatica was reported in 17 patients treated with pembrolizumab; 13 (76%) of these were in recovery or remission, while three (18%) were not. Polymyalgia rheumatica was reported in 12 patients treated with ipilimumab; seven (58%) of these were in recovery or remission. Our study highlights that careful monitoring for the symptom of PMR (e.g., bilateral pain in shoulder and pelvic girdles) is required when the patients are aged >70 years and have been treated with nivolumab and when the patients are women and have been treated with pembrolizumab.

## Introduction

Immune checkpoint inhibitors (ICPIs) are used for chemotherapy in various types of cancers (Hodi et al., 2010; Brahmer et al., 2012; Borghaei et al., 2015; Robert et al., 2015b; Motzer et al., 2015). The pharmacological mechanisms of ICPIs include blockade of programmed cell death 1 (PD-1)/PD ligand 1 (PD-L1) signaling (Brahmer et al., 2010; Herbst et al., 2014) and cytotoxic T-lymphocyte antigen 4 (CTLA-4) signaling (Melero et al., 2007) to activate T cell-mediated antitumor immunity. ICPIs induce immune-related adverse events (irAEs) such as skin disorders (Robert et al., 2015a), gastrointestinal disorders (Robert et al., 2015b), thyroid dysfunction (Robert et al., 2015b; Tie et al., 2017), type 1 diabetes mellitus (DM) (Robert et al., 2015b), lupus nephritis (Fadel et al., 2009), and vasculitides (Daxini et al., 2018). These irAEs are effectively treated by temporary administration of glucocorticoids or additional immunosuppressants in severe cases (Postow et al., 2018; Mamlouk et al., 2020). In addition, an anti-PD-1 therapy can improve the prognosis of patients with severe irAEs induced by ipilimumab (Brunot et al., 2020). However, these therapeutic interventions may decrease the efficacy of chemotherapy, and the use of immunosuppressive agents can lead to some irAEs (Postow et al., 2018).

Identification of the risk factors of irAEs can facilitate their prevention after ICPI chemotherapy. Changes in the composition of the gastrointestinal microflora (Sivan et al., 2015; Vétizou et al., 2015; Chaput et al., 2017), a high body mass index (Guzman-Prado et al., 2021), female sex, and a history of melanoma (Takada et al., 2020) have been identified as risk factors of irAEs. As the frequency of vasculitides is lower than that of other irAEs, identifying the predictors of vasculitides requires analyses based on large-scale databases. The Japanese Adverse Drug Event Report (JADER) database is an open-access repository of adverse drug events (ADEs) that has been maintained by the Pharmaceuticals and Medical Devices Agency since 2012. The JADER database is used to identify predictors of irAEs. For instance, female sex and a history of melanoma have been identified as the risk factors of type 1 diabetes mellitus after ICPI chemotherapy using this database (Takada et al., 2020).

Vasculitides are a group of heterogeneous autoimmune inflammatory diseases that often result in organ injuries. Because the frequency of vasculitides induced by ICPIs is lower than that of other irAEs, they have not been described extensively before (Daxini et al., 2018). Although the previous report suggested that pre-existed giant cell arteritis (GCA) was associated with irAEs (Leonardi et al., 2018; Klavdianou et al., 2021), the information on vasculitides is limited and the predictors of ICPI-induced vasculitides have not been identified. In the present study, we aimed to identify these predictors using the JADER database and to broaden our knowledge of the underlying risk factors and frequency of ICPI-induced vasculitides.

## Materials and Methods

### Data Source and Study Design

Data deposited between April 2004 and March 2020 were extracted from the JADER database. The database comprises three data tables, namely DEMO, DRUG, and REAC. The DEMO file contains data on parameters such as sex and age; the DRUG file contains data on the generic name and route or dates of administration; and the REAC file contains data on the ADEs, their date of occurrence, and outcomes (recovery, remission, no recovery, death, after-effects, and unknown). Based on their contribution to the ADEs, the medications administered were classified into three categories, namely “suspected medicine,” “concomitant medicine,” and “interaction.” The “suspected medicine” category was defined as having caused the ADEs in the present study. Duplicate data from the DRUG and REAC tables were removed, and the DEMO table was linked to the DRUG and REAC tables by using each case identified in the data tables.

Cases without data on the sex or age in the DEMO table were excluded from the dataset. To analyze the association with patients classified in 10-years age intervals, we defined “older adults” as those in their 70, 80, 90, and 100 s according to a previous report (Sugawara et al., 2019). Nivolumab, pembrolizumab, ipilimumab, atezolizumab, durvalumab, and avelumab were selected as the suspected drugs for vasculitides.

### Definition of Patients With Cancer

The primary disease in the HIST tables extracted from the JADER database was defined based on the preferred terms (PTs) in the Medical Dictionary for Regulatory Activities (MedDRA) version 23.1. MedDRA is an internationally used set of terms relating to medical conditions. Essentially, these PTs define the medical condition of the patient. Cancer as primary disease was defined by the PTs after removing duplicate data (see Supplementary Table S1). Cancers that appeared as primary diseases but were not included in Supplementary Table S1 were classified as others/uncertain.

### Definition of Immune Checkpoint Inhibitors and Vasculitides as irAEs

The ADEs in the REAC table were coded according to the PTs in the MedDRA. Vasculitides as irAEs were defined according to the recommendations of the 2012 revised International Chapel Hill Consensus Conference Nomenclature of Vasculitides (Sunderkötter et al., 2018). The PTs for vasculitides are listed in Table 1. When there were multiple occurrences of different vasculitides in same patients, we counted the each events. We counted the onset day of vasculitides since the first exposure of ICPIs.

**TABLE 1:** Preferred terms of vasculitides.

Preferred terms number	Preferred terms
10050894	Anti-neutrophil cytoplasmic antibody positive vasculitis
10002921	Aortitis
10003230	Arteritis
10003232	Arteritis coronary
10004213	Behcet’s syndrome
10068406	Capillaritis
10081778	Central nervous system vasculitis
10008087	Cerebral arteritis
10072726	Chronic pigmented purpura
10056667	Cogan’s syndrome
10011686	Cutaneous vasculitis
10012978	Diffuse vasculitis
10078117	Eosinophilic granulomatosis with polyangiitis
10015213	Erythema induratum
10072579	Granulomatosis with polyangiitis
10071252	Haemorrhagic vasculitis
10019617	Henoch-Schonlein purpura
10069440	Henoch-Schonlein purpura nephritis
10020764	Hypersensitivity vasculitis
10023320	Kawasaki’s disease
10069698	Langerhans’ cell histiocytosis
10058143	Lupus vasculitis
10078132	MAGIC syndrome
10063344	Microscopic polyangiitis
10066926	Ocular vasculitis
10036024	Polyarteritis nodosa
10036099	Polymyalgia rheumatica
10037457	Pulmonary vasculitis
10038373	Renal arteritis
10038546	Renal vasculitis
10038905	Retinal vasculitis
10048628	Rheumatoid vasculitis
10043097	Takayasu’s arteritis
10043207	Temporal arteritis
10043540	Thromboangiitis obliterans
10048820	Urticarial vasculitis
10047097	Vascular purpura
10047111	Vasculitic rash
10047115	Vasculitis
10048319	Vasculitis gastrointestinal
10047124	Vasculitis necrotising
10011474	Cryoglobulinemic vasculitis
10018250	Giant cell arteritis
10014957	Eosinophilic granulomatous vasculitis
10028890	Necrotizing granulomatous vasculitis
10001714	Allergic granulomatosis angiitis
10047888	Wegener’s granulomatosis
10072580	Granulomatous polyangiitis
10082959	IgA vasculitis
10081981	Anti-glomerular basement membrane disease
10077509	Hypocomplementemic urticarial vasculitis syndrome

MAGIC, Mouth and genital ulcers with inflamed cartilage syndrome.

### Statistical Analysis

The reporting odds ratio (ROR), which evaluates the AE signals, was calculated using the following formula (Sugawara et al., 2019):

ROR=(a/b)(c/d)=(a×d)/(b×c),

where a represents number of cases with an ADE related to the use of the suspected drugs; b, number of cases with an ADE related to the use of all other drug; c, number of cases with all other ADEs related to the use of the suspected drug; and d, number of cases with all other ADEs related to the use of all other drugs.

AE signals were considered significant when the ROR estimates and the lower limits of the corresponding 95% confidence interval (CI) exceeded 1. RORs were calculated using Excel for Microsoft 365 (Microsoft Corporation, Redmond, WA, United States).

As polymyalgia rheumatica (PMR) was the most frequent in this study, a chi-square test as univariate analysis and multiple logistic regression analysis were performed to identify the predictors of PMR in patients treated with nivolumab, pembrolizumab, and ipilimumab. Sex and age (≥70 years) were used as variables in the univariate and multivariate analyses. The Hosmer–Lemeshow statistical test was used for testing the goodness of fit for the logistic regression models. A two-sided *p*-value lower than 0.05 was considered statistically significant. The chi-square test, Mann-Whitney U test, multiple logistic regression analysis, and Hosmer–Lemeshow statistical test were performed using SPSS version 22.0 (SPSS, Inc., Chicago, IL, United States).

### Ethics Approval and Consent to Participate

As this study involved an open-access database, ethics approval and consent to participate were not required.

## Results

### Patient Characteristics and the Reporting Odds Ratio Estimates

**FIGURE 1:**
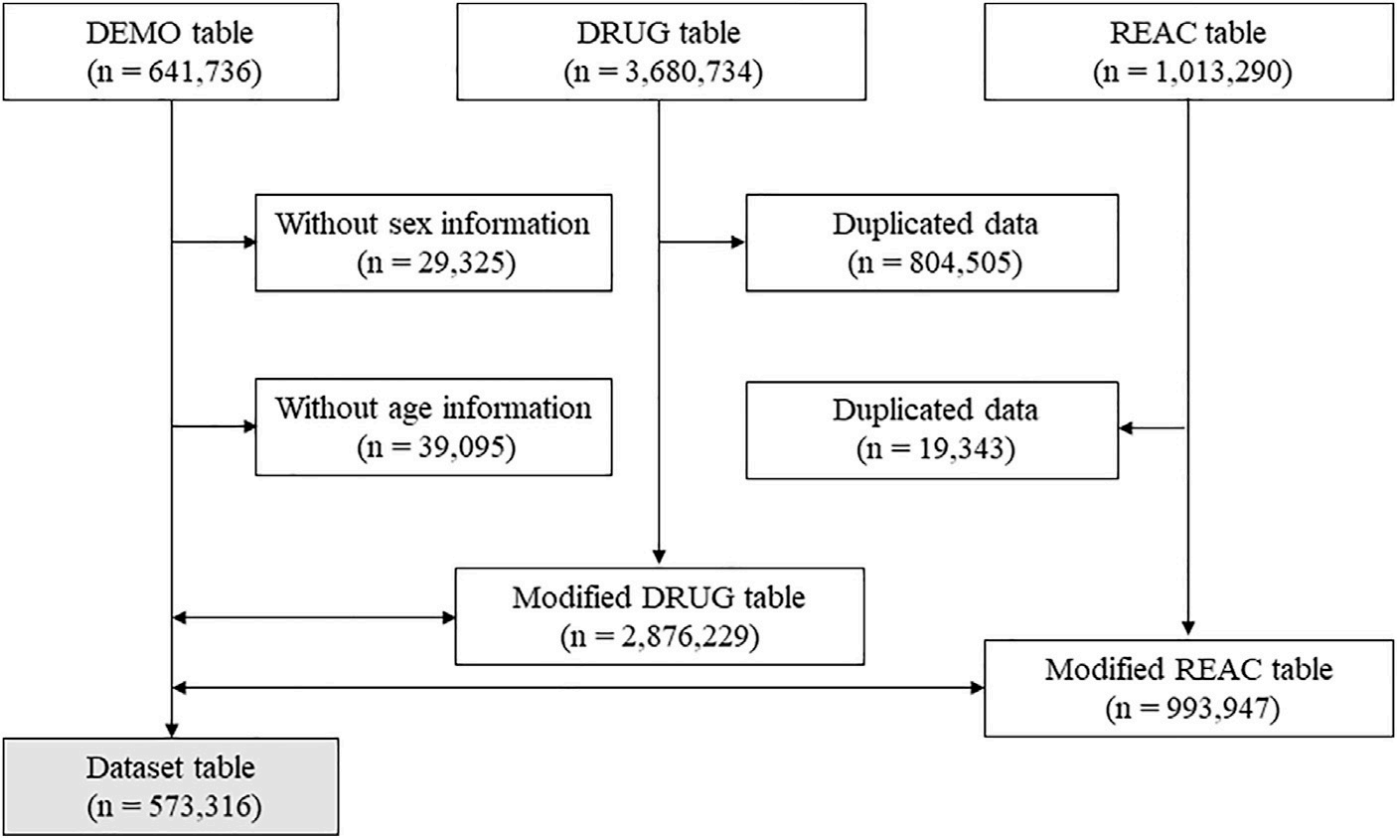
Flow diagram of the study. Dotted arrow and double arrow show data exclusion and combination, respectively.

A total of 573,316 cases were included in the dataset table (Figure 1). Of these, 8,705, 5,202, 2,362, 1,039, 876, and 17 cases had the use of nivolumab, pembrolizumab, ipilimumab, atezolizumab, durvalumab, and avelumab, respectively (Table 2). Characteritics of patients with vasculitides and PMR were shown in Table 3 and Table 4, respectively. The nivolumab, pembrolizumab, atezolizumab, and durvalumab groups predominantly included patients with lung cancer, whereas the ipilimumab group mostly included patients with melanoma (Table 2). ADE signals of vasculitides were detected in the nivolumab, pembrolizumab, ipilimumab, atezolizumab, and durvalumab groups. The use of nivolumab showed a significant signal for vasculitides [ROR (95% CI): 1.498 (1.12–2.00)] (Table 5). The pembrolizumab, ipilimumab, atezolizumab, and durvalumab groups also showed ADE signals for vasculitides, although these were not significant (Table 5). Moreover, the avelumab group showed no ADE signals for vasculitides because of the small sample size.

**TABLE 2:** Patient characteristics.

	Anti PD-1		Anti CTLA-4	Anti PD-L1		
	Nivolumab	Pembrolizumab	Ipilimumab	Atezolizumab	Durvalumab	Avelumab
Total patients (n)	8,705	5,202	2,362	1,039	876	17
Sex Male (n)	6,306	3,969	1,547	756	704	8
Age ≧70 years old (n)	3,812	2,879	942	519	462	13
Cancer type (with overlaps)						
Non-small cell lung cancer (n)	3,244	3,245	3	782	868	0
Head and neck cancer (n)	564	2	2	0	0	0
Urothelial cancer (n)	27	977	4	2	7	0
Renal cell carcinoma (n)	1811	28	991	1	4	0
Melanoma (n)	1,560	125	1,042	1	0	0
Gastric cancer (n)	895	51	6	3	5	0
Hodgkin lymphoma (n)	131	29	0	0	0	0
Mesothelioma (n)	74	1	2	0	0	0
Myeloma (n)	5	3	1	0	0	0
Merkel cell carcinoma (n)	0	1	0	0	0	0
Others/uncertain (n)	479	783	323	253	6	17

PD-1, Programmed cell death 1; PD-L1, Programmed cell death ligand 1; CTLA-4, Cytotoxic T-lymphocyte antigen 4.

**TABLE 3:** Characteristics of patient with vasculitides.

	Anti PD-1		Anti CTLA-4	Anti PD-L1		
	Nivolumab	Pembrolizumab	Ipilimumab	Atezolizumab	Durvalumab	Avelumab
Total patients (n)	48	20	13	3	1	0
Sex Male (n)	39	10	10	3	0	0
Age ≧70 years old (n)	38	14	12	1	1	0
Cancer type (with overlaps)						
Non-small cell lung cancer (n)	14	14	0	3	1	0
Head and neck cancer (n)	4	0	0	0	0	0
Urothelial cancer (n)	0	5	0	0	0	0
Renal cell carcinoma (n)	11	1	8	0	0	0
Melanoma (n)	9	0	4	0	0	0
Gastric cancer (n)	8	0	0	0	0	0
Hodgkin lymphoma (n)	1	1	0	0	0	0
Mesothelioma (n)	1	0	0	0	0	0
Myeloma (n)	0	0	0	0	0	0
Merkel cell carcinoma (n)	0	0	0	0	0	0
Others/uncertain (n)	1	0	1	0	0	0

PD-1, Programmed cell death 1; PD-L1, Programmed cell death ligand 1; CTLA-4, Cytotoxic T-lymphocyte antigen 4.

**TABLE 4:** Characteristics of patient with polymyalgia rheumatic.

	Anti PD-1		Anti CTLA-4	Anti PD-L1		
	Nivolumab	Pembrolizumab	Ipilimumab	Atezolizumab	Durvalumab	Avelumab
Total patients (n)	38	17	12	0	0	0
Sex Male (n)	32	7	10	0	0	0
Age ≧70 years old (n)	35	12	12	0	0	0
Cancer type (with overlaps)						
Non-small cell lung cancer (n)	9	11	0	0	0	0
Head and neck cancer (n)	3	0	0	0	0	0
Urothelial cancer (n)	0	5	0	0	0	0
Renal cell carcinoma (n)	10	1	8	0	0	0
Melanoma (n)	7	0	3	0	0	0
Gastric cancer (n)	7	0	0	0	0	0
Hodgkin lymphoma (n)	0	1	0	0	0	0
Mesothelioma (n)	1	0	0	0	0	0
Myeloma (n)	0	0	0	0	0	0
Merkel cell carcinoma (n)	0	0	0	0	0	0
Others/uncertain (n)	1	0	1	0	0	0

PD-1: Programmed cell death 1, PD-L1: Programmed cell death ligand 1, CTLA-4: Cytotoxic T-lymphocyte antigen 4.

**TABLE 5:** Reporting odds ratios for vasculitides.

ICPI	Vasculitides (*n*)	Others (*n*)	ROR	95%CI
Nivolumab	48	8,657	1.498	1.12–2.00
Pembrolizumab	20	5,182	1.035	0.67–1.61
Ipilimumab	13	2,349	1.487	0.86–2.57
Atezolizumab	3	1,036	0.776	0.25–2.41
Durvalumab	1	875	0.306	0.04–2.17
Avelumab	0	17	N.A.	N.A.

ICPI, Immune checkpoint inhibitor; ROR, Reporting odds ratio; CI, Confidence interval; N.A., Not available.

The number of cases with PMR was the highest among all cases with ICPI-induced vasculitides (PMR: 67 cases, vasculitis: 8 cases, Henoch–Schönlein purpura: 3 cases, temporal arteritis: 2 cases, anti-neutrophil cytoplasmic antibody-positive vasculitis: 1 case, aortitis: 1 case, eosinophilic granulomatosis with polyangiitis: 1 case, Henoch–Schönlein purpura nephritis: 1 case, and retinal vasculitis: 1 case). To avoid the overlooks of PMR cases associated with vasculitides in spontaneous reports, the JADER database classifies PMR cases to the category of vasculitides. Therefore, these cases were detected as vasculitides-related PMR. Although physicians reported all cases to JADER database according to classification of MedDRA, some cases could not be categorized to typical vasculitides. These cases were detected as “vasculitis.” The use of nivolumab, pembrolizumab, and ipilimumab showed significant signals for PMR [ROR (95% CI); nivolumab: 28.78 (19.64–42.17), pembrolizumab: 17.40 (10.43–29.06), and ipilimumab: 26.03 (14.33–47.27)]. There were no reports of PMR in patients treated with atezolizumab, durvalumab, and avelumab. A significant signal of retinal vasculitis after the use of pembrolizumab was also identified [ROR (95% CI): 13.65 (1.71–109.1)]. No significant signals were obtained for other vasculitides (Table 6). To evaluate whether an unknown factor have induced a variation in the occurrence of vasculitis, we compared the numbers of vasculitides from 2004 to 2013 and from 2014 to 2019. There were no significant differences between two groups (Supplementary Figure S1).

**TABLE 6:** Reporting odds ratios for the types of vasculitides.

Terms of vasculitides and ICPI	Vasculitides (*n*)	Others (*n*)	ROR	95%CI
Anti-neutrophil cytoplasmic antibody positive vasculitis				
Nivolumab	1	8,704	0.170	0.02–1.21
Pembrolizumab	0	5,202	N.A.	N.A.
Ipilimumab	0	2,362	N.A.	N.A.
Atezolizumab	0	1,039	N.A.	N.A.
Durvalumab	0	876	N.A.	N.A.
Avelumab	0	17	N.A.	N.A.
Aortitis				
Nivolumab	0	8,705	N.A.	N.A.
Pembrolizumab	1	5,201	1.071	0.15–7.68
Ipilimumab	0	2,362	N.A.	N.A.
Atezolizumab	0	1,039	N.A.	N.A.
Durvalumab	0	876	N.A.	N.A.
Avelumab	0	17	N.A.	N.A.
Eosinophilic granulomatosis with polyangiitis				
Nivolumab	1	8,704	0.351	0.05–2.50
Pembrolizumab	0	5,202	N.A.	N.A.
Ipilimumab	0	2,362	N.A.	N.A.
Atezolizumab	0	1,039	N.A.	N.A.
Durvalumab	0	876	N.A.	N.A.
Avelumab	0	17	N.A.	N.A.
Henoch-Schönlein purpura				
Nivolumab	2	8,703	0.399	0.01–1.60
Pembrolizumab	1	5,201	0.335	0.05–2.38
Ipilimumab	0	2,362	N.A.	N.A.
Atezolizumab	0	1,039	N.A.	N.A.
Durvalumab	0	876	N.A.	N.A.
Avelumab	0	17	N.A.	N.A.
Henoch-Schönlein purpura nephritis				
Nivolumab	1	8,704	2.703	0.37–19.98
Pembrolizumab	0	5,202	N.A.	N.A.
Ipilimumab	0	2,362	N.A.	N.A.
Atezolizumab	0	1,039	N.A.	N.A.
Durvalumab	0	876	N.A.	N.A.
Avelumab	0	17	N.A.	N.A.
Polymyalgia rheumatica				
Nivolumab	38	8,667	28.781	19.64–42.17
Pembrolizumab	17	5,185	17.405	10.43–29.06
Ipilimumab	12	2,350	26.026	14.33–47.27
Atezolizumab	0	1,039	N.A.	N.A.
Durvalumab	0	876	N.A.	N.A.
Avelumab	0	17	N.A.	N.A.
Retinal vasculitis				
Nivolumab	0	8,705	N.A.	N.A.
Pembrolizumab	1	5,201	13.654	1.71–109.19
Ipilimumab	0	2,362	N.A.	N.A.
Atezolizumab	0	1,039	N.A.	N.A.
Durvalumab	0	876	N.A.	N.A.
Avelumab	0	17	N.A.	N.A.
Temporal arteritis				
Nivolumab	1	8,704	4.054	0.54–30.57
Pembrolizumab	0	5,202	N.A.	N.A.
Ipilimumab	0	2,362	N.A.	N.A.
Atezolizumab	1	1,038	34.457	4.57–260.07
Durvalumab	0	876	N.A.	N.A.
Avelumab	0	17	N.A.	N.A.
Vasculitis				
Nivolumab	4	8,701	0.831	0.31–2.23
Pembrolizumab	0	5,202	N.A.	N.A.
Ipilimumab	1	2,361	0.767	0.18–5.47
Atezolizumab	2	1,037	3.513	0.87–14.13
Durvalumab	1	875	2.076	0.29–14.80
Avelumab	0	17	N.A.	N.A.

ICPI, Immune checkpoint inhibitor; ROR, Reporting odds ratio; CI, Confidence interval; N.A., Not available.

### Predictors of Immune Checkpoint Inhibitors-Induced Polymyalgia Rheumatica

Univariate analysis revealed that the frequency of nivolumab-induced PMR was significantly higher in patients aged ≥70 years (older adults) than in patients aged <70 years [OR (95% CI): 15.11 (4.642–49.15), *p* < 0.001]. The frequency of pembrolizumab-induced PMR was significantly lower in male patients than in female patients [OR (95% CI): 0.216 (0.082–0.569), *p* = 0.001]. Multivariate analysis confirmed the results of the univariate analysis. The frequency of nivolumab-induced PMR was significantly higher in older adults (aged ≥70 years) than in those aged <70 years [OR (95% CI): 15.02 (4.615–48.87), *p* < 0.001; Hosmer–Lemeshow test, *p* = 0.711], while the frequency of pembrolizumab-induced PMR was significantly lower in male patients than in female patients [OR (95% CI): 0.214 (0.081–0.564), *p* = 0.002; Hosmer–Lemeshow test, *p* = 0.548] (Table 7). Because only one case of retinal vasculitis was available, multivariate analysis could not be performed for all variables.

**TABLE 7:** Univariate and multivariate analysis for predictors of ICPI-induced polymyalgia rheumatica.

	Univariate analysis Or (95%CI)	*p* Value	Multivariate analysis Or (95%CI)	*p* Value
Nivolumab				
Male	2.034 (0.850–4.871)	0.104	1.985 (0.828–4.759)	0.124
Older adults (≥70 years old)	15.11 (4.642–49.149)	<0.001	15.02 (4.615–48.87)	<0.001
Pembrolizumab				
Male	0.216 (0.082–0.569)	0.001	0.214 (0.081–0.564)	0.002
Older adults (≥70 years old)	1.940 (0.683–5.516)	0.205	1.980 (0.696–5.635)	0.200
Ipilimumab				
Male	2.645 (0.578–12.10)	0.193		
Older adults (≥70 years old)	N.A.	N.A.		

CI, Confidence interval, N.A., Not available.

### Time of Occurrence and Outcomes of Polymyalgia Rheumatica

PMR was reported in 38 patients treated with nivolumab; among these, 12 (32%) experienced the onset within 60 days from the exposure to nivolumab. The date of occurrence was unknown for the remaining 16 (42%) cases. In addition, 31 (82%) cases were in recovery or remission (Table 8). PMR was also reported in 17 patients treated with pembrolizumab; six (35%) of these experienced the onset within 60 days from the exposure to pembrolizumab, whereas two (12%) experienced the onset at over 121 days after the exposure. The date of occurrence was unknown in nine (53%) cases. Furthermore, 13 (76%) cases were in recovery or remission, while three (18%) were not (Table 8). Finally, PMR was reported in 12 patients treated with ipilimumab; of these, four (33%) experienced the onset within 7 days from the exposure to ipilimumab. The date of occurrence was unknown in six (50%) cases, while seven (58%) cases were in recovery or remission (Table 8).

**TABLE 8:** Time-to-onset and outcomes of occurrence of polymyalgia rheumatica.

ICPI	Date of occurrence of polymyalgia rheumatica	*n*	Outcomes	*n*
Nivolumab	Within 7 days	7	Recovery	8
	8–30 days	1	Remission	23
	31–60 days	4	No recovery	0
	61–120 days	6	Death	0
	Over 121 days	4	After-effects	0
	Unknown	16	Unknown	7
Pembrolizumab	Within 7 days	2	Recovery	5
	8–30 days	2	Remission	8
	31–60 days	2	No recovery	3
	61–120 days	0	Death	0
	Over 121 days	2	After-effects	0
	Unknown	9	Unknown	1
Ipilimumab	Within 7 days	4	Recovery	2
	8–30 days	0	Remission	5
	31–60 days	0	No recovery	0
	61–120 days	2	Death	0
	Over 121 days	0	After-effects	0
	Unknown	6	Unknown	5

ICPI: Immune checkpoint inhibitor.

## Discussion

The frequency of vasculitides induced by ICPIs is much lower than that of other irAEs. Although the mortality associated with vasculitides is high (Isobe et al., 2020), the mechanism underlying the development of ICPI-induced vasculitides remains elusive. Determining the underlying molecular mechanisms and identifying the predictors of ICPI-induced vasculitides may improve the prognosis of such patients. Herein, we investigated the predictors of ICPI-induced vasculitides by analyzing real-world data obtained from the JADER database. The use of nivolumab was found to increase the frequency of vasculitides, and PMR was the most frequently reported vasculitis among patients treated with nivolumab, pembrolizumab, and ipilimumab.

PMR is characterized by GCA and inflammatory symptoms, such as aching and morning stiffness in the cervical region (Salvarani et al., 2002). Prednisone treatment at a dose of 12.5–25 mg/day is typically recommended for patients with PMR (Hernández-Rodríguez et al., 2009; Dejaco et al., 2015). However, specific diagnostic markers for PMR are currently lacking, making it difficult to distinguish PMR from similar inflammatory diseases, such as myositis and rheumatoid arthritis. The European League Against Rheumatism/American College of Rheumatology guidelines state that other diagnoses should be excluded at the discretion of the physician. They recommend extensive serological tests, such as tests for anti-nuclear antibodies, anti-cytoplasmic neutrophil antibodies, and tuberculosis, to enable an accurate diagnosis (Dejaco et al., 2015). Because diagnosing PMR is a time-consuming process, withdrawal of drugs suspected to have caused it and diagnostic treatment are commenced in some cases to improve outcomes. Angelopoulou et al. (2021) reviewed clinical studies and case series and reported the frequency of ICPI-induced irAEs. The prevalence rate of musculoskeletal events was reported to be 6.13%, wherein PMR accounted for 12.12% of these events. In addition, the rate of patients who discontinued the treatment because of musculoskeletal conditions reached 19%. Therefore, to prevent discontinuation of ICPI or additional treatments (e.g., steroids and disease modifying anti-rheumatic drugs), the risk factors for musculoskeletal events, including PMR, must be identified.

PD-1 regulates Tcell-mediated autoimmune diseases, and hence, disruption of the PD-1/PD-L1 or PD-L2 axis can trigger PMR. The expression of PD-1 is low in Asian patients with rheumatoid arthritis (Kong et al., 2005; Li et al., 2014). In addition, ICPI-induced PMR has been described in some case reports (Belkhir et al., 2017; Bernier et al., 2017; Nakamagoe et al., 2017; Betrains and Blockmans, 2019; Imai et al., 2019; Lobo et al., 2020; Kita et al., 2021) and clinical reviews (Benfaremo et al., 2018; Angelopoulou et al., 2021). Our findings indicated that blockade of PD-1 or CTLA-4 increases the risk of PMR; this is in line with the reports of previous studies. Because the type of vasculitis more prevalent in patients treated with ICPIs is vaguely understood, the present study provides a new strategy for preventing irAEs in such patients. In addition, older and female patients experienced higher frequencies of nivolumab- and pembrolizumab-induced PMR, respectively. A previous report indicated that the mean age at which ICPI-associated PMR occurred was 75 years, and the frequency of PMR was higher in men than in women (Salem et al., 2018). Because the risk of PMR for each ICPI has not been determined, we could not compare the previous results with ours. However, both our study and the above-mentioned previous study suggest that the frequency of PMR is higher in older patients; further studies are needed to clarify the reasons underlying sex-based differences.

The incidence of PMA peaks at around 70 years of age, and two-thirds of the patients are women (Matteson and Dejaco, 2017). Thus, our findings suggest that the risk factors for PMR are the same as those for PMR unrelated to ICIPs. Belkhir et al. (2017) reported four cases of ICPI-induced PMR. Among these, two experienced PMR onset after treatment with nivolumab or a combination of nivolumab and ipilimumab, while one experienced onset after pembrolizumab treatment. In all three cases, the onset occurred within 120 days from the anti-PD-1 or anti-CTLA-4 therapy. In addition, all cases responded to corticosteroids (Belkhir et al., 2017). Although none of the cases experienced an onset of PMR after an anti-PD-L1 therapy, these findings support our results. In the current study, three patients with pembrolizumab-induced PMR did not recover, suggesting that patients treated with pembrolizumab are at a risk of severe PMR. Because we used databases reporting spontaneous ADEs, the detailed clinical background of these three cases was not available. In order to investigate the mortality and response rate to corticosteroids in patients with pembrolizumab-induced PMR, further clinical studies are required. The monitoring for PMR events after ICPIs therapy should be needed in patients with pre-existed PMR (Menzies et al., 2017).

The present study has some limitations. First, because the JADER database is a large spontaneous reporting system, it is subject to various biases, including under- or over-reporting and confounders caused by comorbidities (Abe et al., 2016; Hara et al., 2017; Hasegawa et al., 2017; Hosoya et al., 2017; Sugawara et al., 2019; Takada et al., 2020). Therefore, we could not elucidate whether ICPIs-induced PMR is the idiopathic form of the disease, or whether it should be considered as a new entity. This limitation has been raised in the previous report (Manzo et al., 2020). In addition, pharmacovigilance studies based solely on the JADER database could create the hypothesis for prospective studies. Second, the ADE signals of vasculitides induced by some ICPIs were weak or not detected because of the small sample size. In Japan, nivolumab was approved in 2014, whereas pembrolizumab, ipilimumab, atezolizumab, and durvalumab were approved in 2016, 2015, 2018, and 2018, respectively. As some of these drugs have been recently approved, studies to evaluate the ADE signals between the events of rare vasculitides and the usage of other ICPIs are currently scanty. Third, the characteristics used in the univariate and multivariate analyses were limited because of missing data. However, this large-scale database provided information on infrequent ADEs. Fourth, some patients with PMR have concomitant GCA; however, its incidence is around 20% (Salvarani et al., 1995). Because the Medical Dictionary for Regulatory Activities has included the term “PMR” within the category of vasculitis, we could not classify PMR as with or without GCA. However, it is possible that the physician reported cases of ICPI-induced PMR, because PMR is under the category of vasculitis in the JADER database. To eliminate this limitation, further studies using real world data are required.

In conclusion, based on the predictors of ICPI-induced PMR identified, it is suggested that careful monitoring for the symptom of PMR (e.g., bilateral pain in shoulder and pelvic girdles) is required in patients over 70 years of age treated with nivolumab and female patients treated with pembrolizumab.

## Data Availability

Publicly available datasets were analyzed in this study. This data can be found here: https://www.info.pmda.go.jp/fukusayoudb/CsvDownload.jsp.

## Abbreviations

ICPI, immune checkpoint inhibitor; PD-1, programmed cell death 1; PD-L1, programmed cell death ligand 1; CTLA-4, cytotoxic T-lymphocyte antigen 4; irAE, immune-related adverse event; ADEs, adverse drug events; PT, preferred term; ROR, reporting odds ratio; CI, confidence interval; PMR, polymyalgia rheumatica; GCA, giant cell arteritis.
