# Supplementary material for: Frequency of Immune Checkpoint Inhibitor-Induced Vasculitides: An Observational Study Using Data From the Japanese Adverse Drug Event Report Database
Source: Front Pharmacol. 2022 Mar 25;13:803706. doi: 10.3389/fphar.2022.803706 (PMC8992371; doi:10.3389/fphar.2022.803706)
Supplement: Supplementary file 1 [file DataSheet1.docx]

**Frequency of Immune Checkpoint Inhibitor-induced Vasculitides: An Observational Study Using Data from the Japanese Adverse Drug Event Report** **Database**

Koki Kato^1^, Tomohiro Mizuno^1^**^*^**, Takenao Koseki^1^, Yoshimasa Ito^2^, Kazuo Takahashi^3^, Naotake Tsuboi^2^ and Shigeki Yamada^1^

^1^Department of Clinical Pharmacy, Fujita Health University School of Medicine, Toyoake, Japan

^2^Department of Nephrology, Fujita Health University School of Medicine, Toyoake, Japan

^3^Department of Biomedical Molecular Sciences, Fujita Health University School of Medicine, Toyoake, Japan

**^*^***Corresponding author*: Tomohiro Mizuno, Ph.D.

Department of Clinical Pharmacy, Fujita Health University School of Medicine

1-98 Dengakugakubo, Kutsukake-cho, Toyoake, 470-1192, Japan

Tel: (+81) 562-93-2493

Fax: (+81) 562-93-4593

E-mail: tomohiro.mizuno@fujita-hu.ac.jp

**Supplementary Table 1** Preferred terms of cancer as a primary disease

| Cancer type | Preferred terms number | Preferred terms |
| --- | --- | --- |
| Non-small cell lung cancer | 10001245 | Adenosquamous cell lung cancer |
|  | 10001247 | Adenosquamous cell lung cancer recurrent |
|  | 10001248 | Adenosquamous cell lung cancer stage 0 |
|  | 10001249 | Adenosquamous cell lung cancer stage I |
|  | 10001250 | Adenosquamous cell lung cancer stage II |
|  | 10001251 | Adenosquamous cell lung cancer stage III |
|  | 10001254 | Adenosquamous cell lung cancer stage IV |
|  | 10023775 | Large cell lung cancer recurrent |
|  | 10023776 | Large cell lung cancer stage 0 |
|  | 10023777 | Large cell lung cancer stage I |
|  | 10023778 | Large cell lung cancer stage II |
|  | 10023779 | Large cell lung cancer stage III |
|  | 10023780 | Large cell lung cancer stage IV |
|  | 10025031 | Lung adenocarcinoma |
|  | 10025033 | Lung adenocarcinoma recurrent |
|  | 10025034 | Lung adenocarcinoma stage 0 |
|  | 10025035 | Lung adenocarcinoma stage I |
|  | 10025036 | Lung adenocarcinoma stage II |
|  | 10025037 | Lung adenocarcinoma stage III |
|  | 10025038 | Lung adenocarcinoma stage IV |
|  | 10025120 | Lung squamous cell carcinoma recurrent |
|  | 10025121 | Lung squamous cell carcinoma stage 0 |
|  | 10025122 | Lung squamous cell carcinoma stage I |
|  | 10025123 | Lung squamous cell carcinoma stage II |
|  | 10025124 | Lung squamous cell carcinoma stage III |
|  | 10025125 | Lung squamous cell carcinoma stage IV |
|  | 10029515 | Non-small cell lung cancer recurrent |
|  | 10029516 | Non-small cell lung cancer stage 0 |
|  | 10029517 | Non-small cell lung cancer stage I |
|  | 10029518 | Non-small cell lung cancer stage II |
|  | 10029519 | Non-small cell lung cancer stage III |
|  | 10029520 | Non-small cell lung cancer stage IIIA |
|  | 10029521 | Non-small cell lung cancer stage IIIB |
|  | 10029522 | Non-small cell lung cancer stage IV |
|  | 10061873 | Non-small cell lung cancer |
|  | 10069730 | Large cell lung cancer metastatic |
|  | 10071533 | Lung squamous cell carcinoma metastatic |
|  | 10059515 | Non-small cell lung cancer metastatic |
| Head and neck cancer | 10071540 | Head and neck cancer metastatic |
|  | 10067821 | Head and neck cancer |
|  | 10071539 | Head and neck cancer stage I |
|  | 10071538 | Head and neck cancer stage II |
|  | 10071537 | Head and neck cancer stage III |
|  | 10071536 | Head and neck cancer stage IV |
|  | 10060121 | Squamous cell carcinoma of head and neck |
| Urothelial cancer | 10004986 | Bladder adenocarcinoma recurrent |
|  | 10004987 | Bladder adenocarcinoma stage 0 |
|  | 10004988 | Bladder adenocarcinoma stage I |
|  | 10004989 | Bladder adenocarcinoma stage II |
|  | 10004990 | Bladder adenocarcinoma stage III |
|  | 10004991 | Bladder adenocarcinoma stage IV |
|  | 10004992 | Bladder adenocarcinoma stage unspecified |
|  | 10005003 | Bladder cancer |
|  | 10005005 | Bladder cancer recurrent |
|  | 10005006 | Bladder cancer stage 0, with cancer in situ |
|  | 10005008 | Bladder cancer stage I, with cancer in situ |
|  | 10005010 | Bladder cancer stage II |
|  | 10005011 | Bladder cancer stage III |
|  | 10005012 | Bladder cancer stage IV |
|  | 10005075 | Bladder squamous cell carcinoma recurrent |
|  | 10005076 | Bladder squamous cell carcinoma stage 0 |
|  | 10005077 | Bladder squamous cell carcinoma stage I |
|  | 10005078 | Bladder squamous cell carcinoma stage II |
|  | 10005079 | Bladder squamous cell carcinoma stage III |
|  | 10005080 | Bladder squamous cell carcinoma stage IV |
|  | 10005081 | Bladder squamous cell carcinoma stage unspecified |
|  | 10005084 | Bladder transitional cell carcinoma |
|  | 10057352 | Metastatic carcinoma of the bladder |
|  | 10066749 | Bladder transitional cell carcinoma stage 0 |
|  | 10066750 | Bladder transitional cell carcinoma recurrent |
|  | 10066751 | Bladder transitional cell carcinoma stage I |
|  | 10066752 | Bladder transitional cell carcinoma stage IV |
|  | 10066753 | Bladder transitional cell carcinoma stage II |
|  | 10066754 | Bladder transitional cell carcinoma stage III |
|  | 10071664 | Bladder transitional cell carcinoma metastatic |
|  | 10078341 | Neuroendocrine carcinoma of the bladder |
|  | 10026426 | Malignant neoplasm of renal pelvis |
|  | 10044406 | Transitional cell cancer of renal pelvis and ureter metastatic |
|  | 10044407 | Transitional cell cancer of the renal pelvis and ureter |
|  | 10044408 | Transitional cell cancer of the renal pelvis and ureter localised |
|  | 10044410 | Transitional cell cancer of the renal pelvis and ureter recurrent |
|  | 10044411 | Transitional cell cancer of the renal pelvis and ureter regional |
|  | 10046392 | Ureteric cancer |
|  | 10046393 | Ureteric cancer local |
|  | 10046394 | Ureteric cancer metastatic |
|  | 10046396 | Ureteric cancer recurrent |
|  | 10046397 | Ureteric cancer regional |
|  | 10026326 | Malignant neoplasm of paraurethral glands |
|  | 10044412 | Transitional cell carcinoma |
|  | 10044426 | Transitional cell carcinoma urethra |
|  | 10046431 | Urethral cancer |
|  | 10046433 | Urethral cancer metastatic |
|  | 10046435 | Urethral cancer recurrent |
|  | 10061272 | Malignant urinary tract neoplasm |
|  | 10061396 | Urinary tract carcinoma in situ |
|  | 10071080 | Transitional cell carcinoma metastatic |
|  | 10074419 | Malignant genitourinary tract neoplasm |
|  | 10077051 | Transitional cell carcinoma recurrent |
|  | 10005056 | Bladder neoplasm |
|  | 10061398 | Urinary tract neoplasm |
|  | 10062221 | Ureteral neoplasm |
|  | 10062223 | Urethral neoplasm |
| Renal cell carcinoma | 10009253 | Clear cell sarcoma of the kidney |
|  | 10029145 | Nephroblastoma |
|  | 10038389 | Renal cancer |
|  | 10038390 | Renal cancer recurrent |
|  | 10038391 | Renal cancer stage I |
|  | 10038392 | Renal cancer stage II |
|  | 10038393 | Renal cancer stage III |
|  | 10038394 | Renal cancer stage IV |
|  | 10038410 | Renal cell carcinoma recurrent |
|  | 10038411 | Renal cell carcinoma stage I |
|  | 10038412 | Renal cell carcinoma stage II |
|  | 10038413 | Renal cell carcinoma stage III |
|  | 10038414 | Renal cell carcinoma stage IV |
|  | 10039019 | Rhabdoid tumour of the kidney |
|  | 10050018 | Renal cancer metastatic |
|  | 10050176 | Renal oncocytoma |
|  | 10050513 | Metastatic renal cell carcinoma |
|  | 10051948 | Renal adenoma |
|  | 10061482 | Renal neoplasm |
|  | 10061872 | Non-renal cell carcinoma of kidney |
|  | 10067943 | Hereditary papillary renal carcinoma |
|  | 10067944 | Hereditary leiomyomatosis renal cell carcinoma |
|  | 10067946 | Renal cell carcinoma |
|  | 10069908 | Renal haemangioma |
|  | 10073251 | Clear cell renal cell carcinoma |
|  | 10078493 | Papillary renal cell carcinoma |
|  | 10080544 | Chromophobe renal cell carcinoma |
|  | 10081895 | Multilocular cystic nephroma |
|  | 10083207 | Renal hamartoma |
| Melanoma | 10025650 | Malignant melanoma |
|  | 10025652 | Malignant melanoma in situ |
|  | 10025668 | Malignant melanoma stage I |
|  | 10025669 | Malignant melanoma stage II |
|  | 10025670 | Malignant melanoma stage III |
|  | 10025671 | Malignant melanoma stage IV |
|  | 10027480 | Metastatic malignant melanoma |
| Gastric cancer | 10001150 | Adenocarcinoma gastric |
|  | 10017758 | Gastric cancer |
|  | 10017761 | Gastric cancer recurrent |
|  | 10017762 | Gastric cancer stage 0 |
|  | 10017763 | Gastric cancer stage I |
|  | 10017764 | Gastric cancer stage II |
|  | 10017765 | Gastric cancer stage III |
|  | 10055008 | Gastric sarcoma |
|  | 10061967 | Gastric cancer stage IV |
|  | 10062878 | Gastrooesophageal cancer |
|  | 10063916 | Metastatic gastric cancer |
|  | 10066896 | HER2 positive gastric cancer |
|  | 10081398 | Gastrooesophageal cancer recurrent |
| Hodgkin lymphoma | 10020208 | Hodgkin's disease lymphocyte depletion stage I site unspecified |
|  | 10020209 | Hodgkin's disease lymphocyte depletion stage I subdiaphragm |
|  | 10020210 | Hodgkin's disease lymphocyte depletion stage I supradiaphragm |
|  | 10020211 | Hodgkin's disease lymphocyte depletion stage II site unspecified |
|  | 10020212 | Hodgkin's disease lymphocyte depletion stage II subdiaphragm |
|  | 10020213 | Hodgkin's disease lymphocyte depletion stage II supradiaphragm |
|  | 10020215 | Hodgkin's disease lymphocyte depletion type recurrent |
|  | 10020216 | Hodgkin's disease lymphocyte depletion type refractory |
|  | 10020217 | Hodgkin's disease lymphocyte depletion type stage III |
|  | 10020218 | Hodgkin's disease lymphocyte depletion type stage IV |
|  | 10020219 | Hodgkin's disease lymphocyte depletion type stage unspecified |
|  | 10020220 | Hodgkin's disease lymphocyte predominance stage I site unspec |
|  | 10020221 | Hodgkin's disease lymphocyte predominance stage I subdiaphragm |
|  | 10020222 | Hodgkin's disease lymphocyte predominance stage I supradiaphragm |
|  | 10020223 | Hodgkin's disease lymphocyte predominance stage II site unspec |
|  | 10020224 | Hodgkin's disease lymphocyte predominance stage II subdiaphragm |
|  | 10020225 | Hodgkin's disease lymphocyte predominance stage II supradiaphragm |
|  | 10020227 | Hodgkin's disease lymphocyte predominance type recurrent |
|  | 10020228 | Hodgkin's disease lymphocyte predominance type refractory |
|  | 10020229 | Hodgkin's disease lymphocyte predominance type stage III |
|  | 10020230 | Hodgkin's disease lymphocyte predominance type stage IV |
|  | 10020231 | Hodgkin's disease lymphocyte predominance type stage unspecified |
|  | 10020206 | Hodgkin's disease |
|  | 10020266 | Hodgkin's disease recurrent |
|  | 10020267 | Hodgkin's disease refractory |
|  | 10020268 | Hodgkin's disease stage I |
|  | 10020269 | Hodgkin's disease stage II |
|  | 10020270 | Hodgkin's disease stage III |
|  | 10020271 | Hodgkin's disease unclassifiable |
|  | 10061597 | Hodgkin's disease stage IV |
|  | 10020233 | Hodgkin's disease mixed cellularity recurrent |
|  | 10020234 | Hodgkin's disease mixed cellularity refractory |
|  | 10020235 | Hodgkin's disease mixed cellularity stage I site unspecified |
|  | 10020236 | Hodgkin's disease mixed cellularity stage I subdiaphragmatic |
|  | 10020237 | Hodgkin's disease mixed cellularity stage I supradiaphragmatic |
|  | 10020238 | Hodgkin's disease mixed cellularity stage II subdiaphragmatic |
|  | 10020239 | Hodgkin's disease mixed cellularity stage II supradiaphragmatic |
|  | 10020240 | Hodgkin's disease mixed cellularity stage III |
|  | 10020241 | Hodgkin's disease mixed cellularity stage IV |
|  | 10020242 | Hodgkin's disease mixed cellularity stage unspecified |
|  | 10020244 | Hodgkin's disease nodular sclerosis |
|  | 10020245 | Hodgkin's disease nodular sclerosis recurrent |
|  | 10020246 | Hodgkin's disease nodular sclerosis refractory |
|  | 10020252 | Hodgkin's disease nodular sclerosis stage III |
|  | 10020253 | Hodgkin's disease nodular sclerosis stage IV |
|  | 10073534 | Hodgkin's disease nodular sclerosis stage II |
|  | 10073535 | Hodgkin's disease nodular sclerosis stage I |
| Mesothelioma | 10027406 | Mesothelioma |
|  | 10027407 | Mesothelioma malignant |
|  | 10027411 | Mesothelioma malignant recurrent |
|  | 10034480 | Pericardial mesothelioma malignant recurrent |
|  | 10034671 | Peritoneal mesothelioma malignant recurrent |
|  | 10035603 | Pleural mesothelioma |
|  | 10035607 | Pleural mesothelioma malignant recurrent |
|  | 10056558 | Peritoneal mesothelioma malignant |
|  | 10059518 | Pleural mesothelioma malignant |
|  | 10073062 | Biphasic mesothelioma |
|  | 10073063 | Desmoplastic mesothelioma |
|  | 10073064 | Epithelioid mesothelioma |
|  | 10073065 | Sarcomatoid mesothelioma |
|  | 10073066 | Pericardial mesothelioma malignant |
| Myeloma | 10035222 | Plasma cell leukaemia |
|  | 10035226 | Plasma cell myeloma |
|  | 10035484 | Plasmacytoma |
|  | 10053869 | POEMS syndrome |
|  | 10060406 | Plasma cell leukaemia in remission |
|  | 10073132 | Plasma cell myeloma in remission |
|  | 10073133 | Plasma cell myeloma recurrent |
|  | 10078282 | Leptomeningeal myelomatosis |
|  | 10081847 | Plasma cell myeloma refractory |
| Merkel cell carcinoma | 10029266 | Neuroendocrine carcinoma of the skin |

HER2: Human epidermal growth factor receptor 2, POEMS: Polyneuropathy, organomegaly, endocrinopathy, m-protein, and skin changes syndrome

**
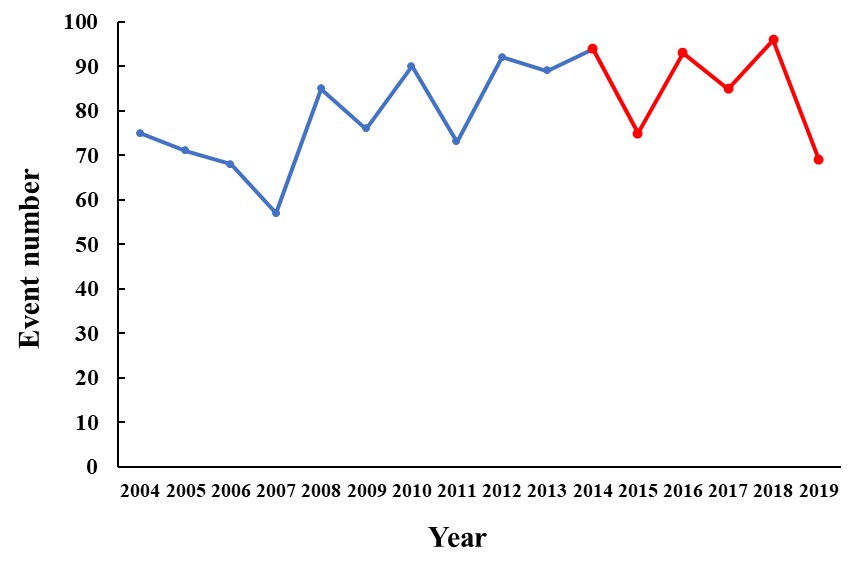
**

**Supplementary Figure 1** Yearly changes in the number of vasculitides event in the JADER database

Blue and red lines show the number of vasculitides event from 2004 to 2013 and from 2014 to 2019, respectively. The median (range) numbers were 76 (57-92) and 85 (69-96) from 2004 to 2013 and from 2014 to 2019, respectively. p = 0.158 (Mann-Whitney U test). JADER: Japanese Adverse Drug Event Report.
